# 3-{(*E*)-[4-(4-Hy­droxy-3-meth­oxy­phen­yl)butan-2-yl­idene]amino}-1-phenyl­urea: crystal structure and Hirshfeld surface analysis

**DOI:** 10.1107/S2056989017017273

**Published:** 2018-01-01

**Authors:** Ming Yueh Tan, Karen A. Crouse, Thahira B. S. A. Ravoof, Mukesh M. Jotani, Edward R. T. Tiekink

**Affiliations:** a Department of Physical Science, Faculty of Applied Sciences, Tunku Abdul Rahman, University College, 50932 Setapak, Kuala Lumpur, Malaysia; b Department of Chemistry, Faculty of Science, Universiti Putra Malaysia, 43400, UPM Serdang, Selangor Darul Ehsan, Malaysia; c Department of Chemistry, St. Francis Xavier University, PO Box 5000, Antigonish, NS B2G 2W5, Canada; d Department of Physics, Bhavan’s Sheth R. A. College of Science, Ahmedabad, Gujarat 380001, India; e Research Centre for Crystalline Materials, School of Science and Technology, Sunway University, 47500 Bandar Sunway, Selangor Darul Ehsan, Malaysia

**Keywords:** crystal structure, urea derivative, hydrogen bonding, Hirshfeld surface analysis

## Abstract

Two independent mol­ecules (*A* and *B*) comprise the asymmetric unit of the title compound, C_18_H_21_N_3_O_3_. The urea moiety is disubstituted with one amine being linked to a phenyl ring, which is twisted out of the plane of the CN_2_O urea core [dihedral angles = 25.57 (11) (*A*) and 29.13 (10)° (*B*)]. The second amine is connected to an imine (*E* conformation), which is linked in turn to an ethane bridge that links a disubstituted benzene ring. Intra­molecular amine-N—H⋯N(imine) and hydroxyl-O—H⋯O(meth­oxy) hydrogen bonds close *S*(5) loops in each case. The mol­ecules have twisted conformations with the dihedral angles between the outer rings being 38.64 (81) (*A*) and 48.55 (7)° (*B*). In the crystal, amide-N—H⋯O(amide) hydrogen bonds link the mol­ecules *A* and *B via* an eight-membered {⋯HNCO}_2_ synthon. Further associations between mol­ecules, leading to supra­molecular layers in the *ac* plane, are hydrogen bonds of the type hydroxyl-O—H⋯N(imine) and phenyl­amine-N—H⋯O(meth­oxy). Connections between layers, leading to a three-dimensional architecture, comprise benzene-C—H⋯O(hy­droxy) inter­actions. A detailed analysis of the calculated Hirshfeld surfaces shows mol­ecules *A* and *B* participate in very similar inter­molecular inter­actions and that any variations relate to conformational differences between the mol­ecules.

## Abstract 2

The disubstituted urea mol­ecule has a twisted conformation for each of the two mol­ecules comprising the asymmetric unit. Intra­molecular amine-N—H⋯N(imine) and hy­droxy-O—H⋯O(meth­oxy) hydrogen bonds are noted. In the mol­ecular packing, amide-N—H⋯O(amide), hydroxyl-O—H⋯N(imine) and phenyl­amine-N—H⋯O(meth­oxy) hydrogen bonding leads to layers in the *ac* plane.

## Chemical context   

Semicarbazones belong to the general class of mol­ecules termed Schiff bases and are prepared from condensation of semicarbazides with aldehydes/ketones. They have attracted considerable attention due to their wide spectrum of bio­logical activities, including anti-convulsant (Pandey & Srivastava, 2010 ▸), anti-tubercular (Sriram *et al.*, 2004 ▸), anti-cancer (Ali *et al.*, 2012 ▸) and anti-microbial (Beraldo & Gambino, 2004 ▸). Actually, they have been investigated extensively for their anti-convulsant properties with 4-(4-fluoro­phen­oxy)benz­aldehyde semicarbazone, in particular, attracting attention as a potent anti-epileptic drug over the past 15 years (Pandeya, 2012 ▸). Recently, the crystal structures of related chalcone-derived thio­semicarbazones and their transition metal complexes have been reported (Tan *et al.*, 2015 ▸, 2017 ▸). In this contribution, aryl semicarbazide is introduced with vanillyl­acetone, which led to the formation of the title compound. Vanillylacetone is one of the active components of ginger and possesses strong anti-oxidant and chemopreventive properties (Kıyak *et al.*, 2015 ▸). The structural elucidation of such compounds has not been extensively investigated. In order to redress this, herein the crystal and mol­ecular structures of the title compound, (I), are described along with an analysis of the calculated Hirshfeld surface in order to ascertain more details of the supra­molecular association operating in the crystal.
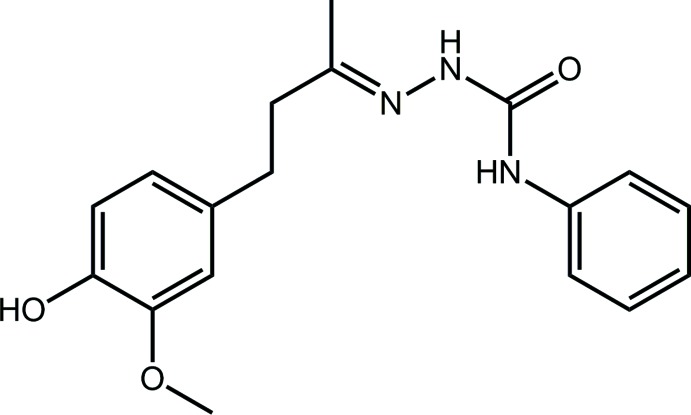


## Structural commentary   

**Figure 1:**
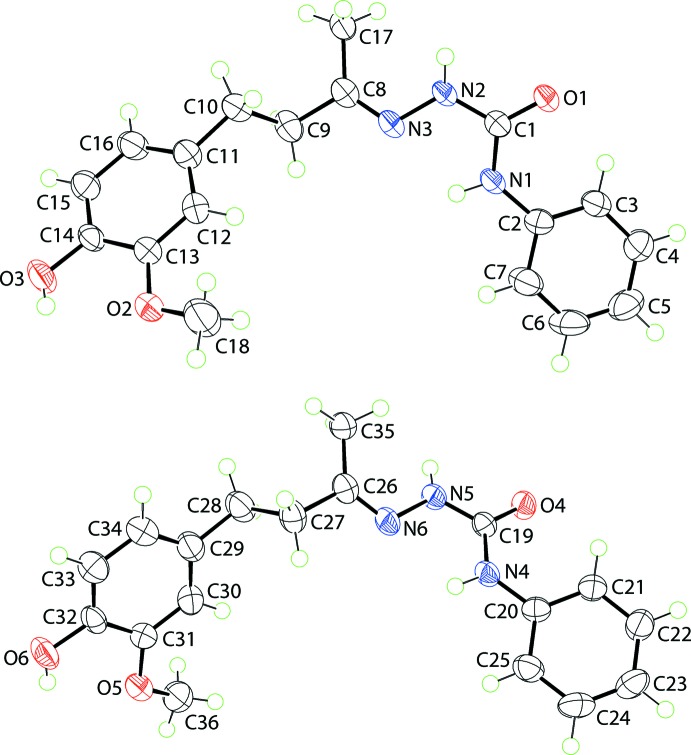
The mol­ecular structures of the two independent mol­ecules comprising the asymmetric unit of (I), showing the atom-labelling scheme and displacement ellipsoids at the 70% probability level.

**Figure 2:**
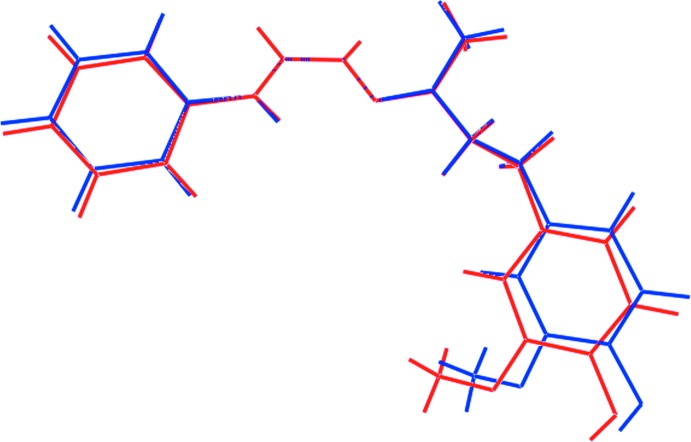
Overlay diagram for (I), with the O1-mol­ecule (red image) and O4-mol­ecule (blue image) superimposed so that the urea residues are coincident.

Two independent mol­ecules, *A* and *B*, comprise the asymmetric unit of (I) and these are shown in Fig. 1 ▸. Each mol­ecule features a disubstituted urea mol­ecule with one amine group connected to a phenyl ring and the other linked to a disubstituted imine group, with the longer side-chain carrying an ethane chain terminating with a disubstituted benzene ring. The four atoms comprising the urea core are strictly planar with an r.m.s. deviation of 0.0041 Å [0.0043 for the O4-mol­ecule, mol­ecule *B*]. The phenyl ring is inclined to this plane, forming a dihedral angle of 25.57 (11)° [29.13 (10)° for mol­ecule *B*]. Intra­molecular N—H⋯N hydrogen bonds are found within the urea residues, Table 1 ▸. A significant kink in the mol­ecule occurs in the ethane bridge, as seen in the value of −157.88 (16)° for the C8—C9—C10—C11 torsion angle [C26—C27—C28—C29 = 162.93 (17)° for *B*]. As a result, the mol­ecule is twisted with the terminal rings inclined to each other, forming a (C2–C7)/(C11–C16) dihedral angle of 38.64 (8)° [(C20–C25)/(C29–C34) = 48.55 (7)° for *B*]. The latter represents the major difference between mol­ecules *A* and *B*, as illustrated in the overlay diagram shown in Fig. 2 ▸. In each of the disubstituted benzene rings, the hydroxyl-H atom is orientated to allow the formation of intra­molecular O—H⋯O hydrogen bonds with the meth­oxy-O atom, Table 1 ▸. The conformation about the imine bond [N3=C8 = 1.281 (2) and N6=C26 = 1.276 (2) Å] is *E* in each mol­ecule. Finally, each of the meth­oxy substituents is twisted out of the plane of the ring to which it is bonded [C18—O2—C13—C12 = 11.7 (3) and C36—O5—C31—C30 = −16.5 (3)°].

## Supra­molecular features   

**Figure 3:**
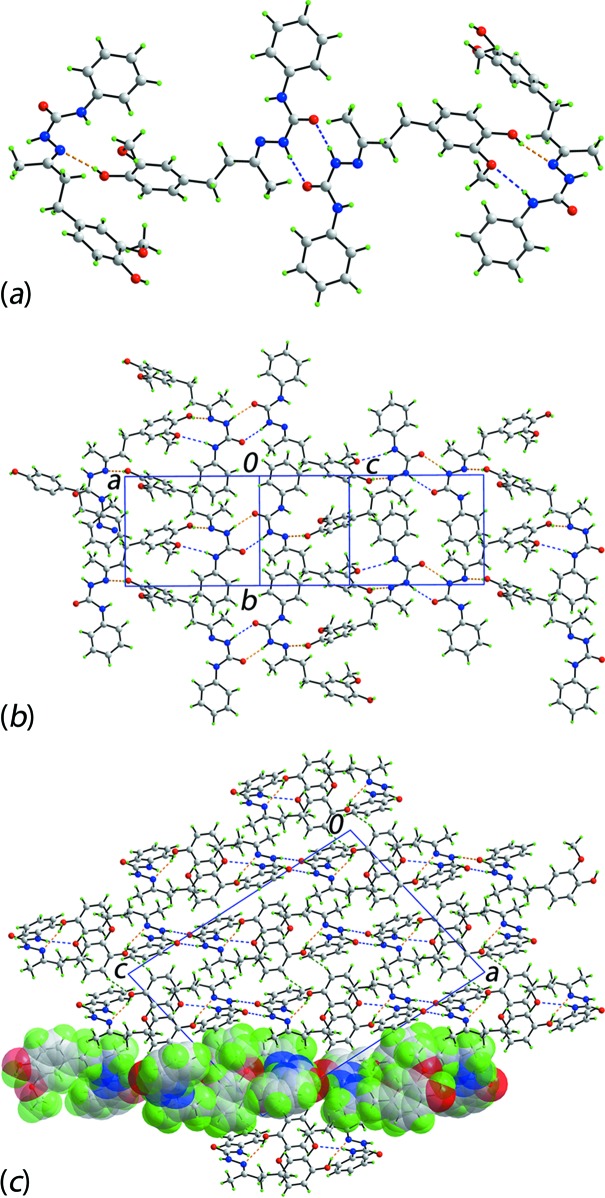
The mol­ecular packing in (I): (*a*) a detail of the supra­molecular association sustained by O—H⋯N and N—H⋯O hydrogen bonding, shown as orange and blue dashed lines, respectively, (*b*) a view of the supra­molecular layer in the *ac* plane, and (*c*) a view of the unit-cell contents shown in projection down the *b* axis. The C—H⋯O inter­actions are shown as green dashed lines, and one layer is highlighted in space-filling mode.

Conventional O—H⋯N and N—H⋯O hydrogen bonding features significantly in the mol­ecular packing of (I), Table 1 ▸, and this is highlighted in Fig. 3 ▸ *a*. The two mol­ecules comprising the asymmetric unit associate *via* an eight-membered amide synthon, {⋯OCNH}_2_. The hy­droxy-O—H groups at each end of the dimeric aggregate hydrogen bond to an imine-N atom of the other independent mol­ecule. The hydroxyl-O3—H⋯N6(imine) inter­action is incorporated within a 10-membered {⋯HOC_2_O⋯HNCNN} heterosynthon owing to the formation of a relatively weak phenyl­amine-N4—H⋯O2(meth­oxy) hydrogen bond. The putative phenyl­amine-N1—H⋯O5(meth­oxy) hydrogen bond is beyond the standard limits (Spek, 2009 ▸) as the H⋯O separation is 2.73 Å. As seen in Fig. 3 ▸ *b*, these hydrogen bonds extend laterally to from an array in (101). The most obvious connections between the supra­molecular layers are of the type benzene-C—H⋯O(hydrox­yl), which occur between centrosymmetrically related O6-benzene rings. A view of the unit-cell contents highlighting the stacking of layers is shown in Fig. 3 ▸ *c*. Other C—H⋯O and several C—H⋯π inter­actions occur in the crystal but within the layers stabilized by hydrogen bonding. These and other weak inter­actions are discussed in more detail in *Analysis of the Hirshfeld surface* (§4).

## Analysis of the Hirshfeld surface   

The Hirshfeld surface was calculated for the individual O1- and O4-mol­ecules in (I), *i.e*. mol­ecules *A* and *B*, and for overall (I) in accord with a recent report on a related mol­ecule (Tan *et al.*, 2017 ▸). These calculations provide additional information about the influence of weak inter­molecular C—H⋯O and C—H⋯π inter­actions, Table 1 ▸, along with short inter­atomic H⋯H, C⋯H/H⋯C and O⋯H/H⋯O contacts, Table 2 ▸, on the mol­ecular packing in the crystal.

**Figure 4:**
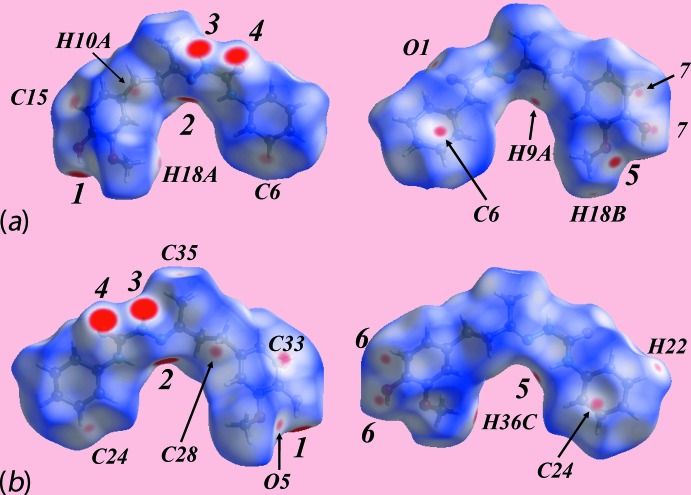
Views of the Hirshfeld surface for (I) mapped over *d*_norm_ in the ranges (*a*) −0.150 to +1.462 au for the O1-containing mol­ecule and (*b*) −0.215 to + 1.462 au for the O4-mol­ecule.

The bright-red spots appearing near the hydroxyl-H3*O* and H6*O*, and imine-N3 and N6 atoms on the Hirshfeld surfaces mapped over *d*_norm_ shown with labels ‘*1*’ and ‘*2*’ in Fig. 4 ▸ represent the donors and acceptors of inter­molecular hydroxyl-O—H⋯N(imine) hydrogen bonds, respectively, Table 1 ▸. In the same way, the prominent red regions near the amide-H2*N* and H5*N*, and amide-O1 and O4 atoms, *i.e*. ‘*3*’ and ‘*4*’ in Fig. 4 ▸, indicate their participation in the inter­molecular N—H⋯O hydrogen bonds between the symmetry-related independent mol­ecules, Table 1 ▸. The donors and acceptors of comparatively weak inter­molecular N—H⋯O and C—H⋯O inter­actions summarized in Table 1 ▸ are viewed as faint-red spots near the respective atoms on *d*_norm_-mapped Hirshfeld surfaces with labels ‘*5*–*7*’ in Fig. 4 ▸.

The presence of diminutive red spots viewed near phenyl atoms C6 in Fig. 4 ▸ *a* and C24 in Fig. 4 ▸ *b*, of the independent mol­ecules, respectively, reflect short inter­atomic edge-to-edge C⋯C contacts, Table 2 ▸, although they contribute a very low contribution, *i.e*. 0.1%, to the Hirshfeld surface owing to the absence of π–π stacking between aromatic rings in the crystal, Table 3 ▸. The faint-red spots appearing near the labelled H10*A*, H18*A*, C28, C6, C33 and C24 atoms in the images of Fig. 4 ▸ represent their participation in short inter­atomic C⋯H/H⋯C contacts, Table 2 ▸, and confirm the influence of the inter­molecular C—H⋯π inter­actions, Table 1 ▸, in the crystal. In addition to these short inter­atomic C⋯H/H⋯C contacts, the faint-red spots near the C15 O1, H9*A* and H18*B* atoms, Fig. 4 ▸ *a*, and O5, C35, H22 and H36*C* atoms, Fig. 4 ▸ *b*, indicate the contributions from the additional short inter­atomic C⋯H/H⋯C and O⋯H/H⋯O contacts, Table 2 ▸, to the mol­ecular packing.

**Figure 5:**
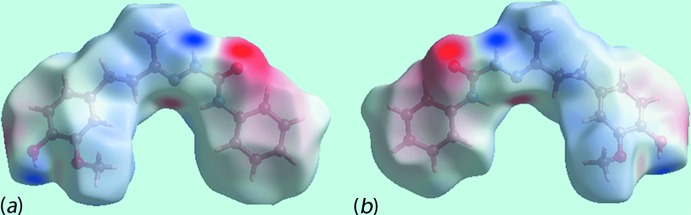
Views of the Hirshfeld surface for (I) mapped over the electrostatic potential in the range −0.103 to + 0.141 au for the (*a*) O1-containing mol­ecule and (*b*) the O4-mol­ecule. The red and blue regions represent negative and positive electrostatic potentials, respectively.

**Figure 6:**
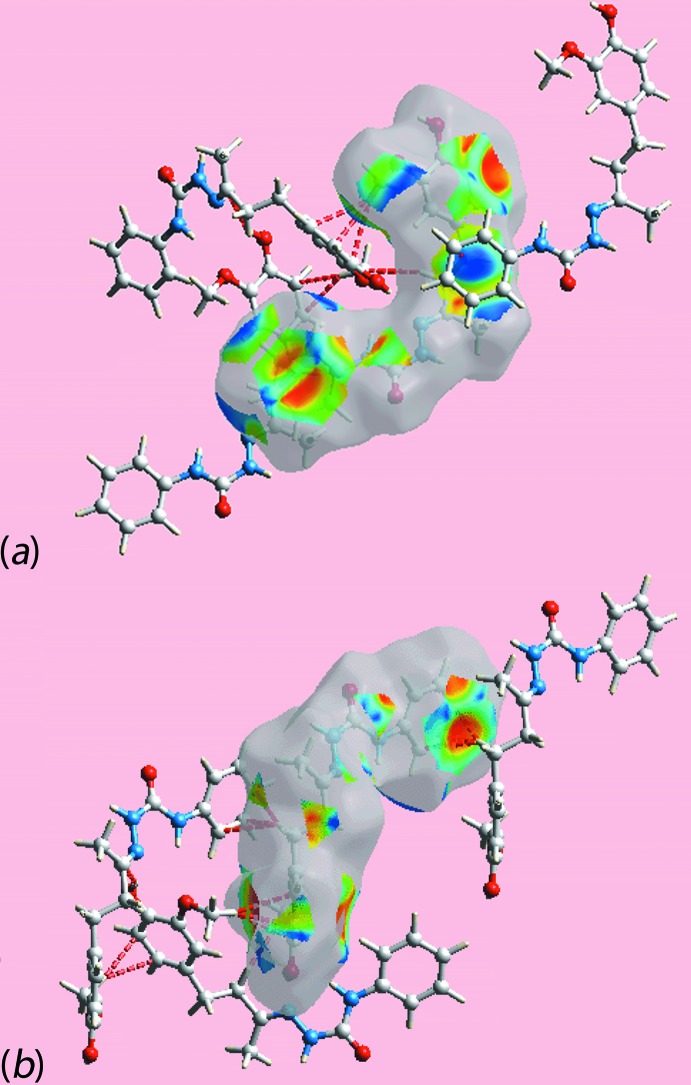
Views of the shape-indexed Hirshfeld surfaces about reference mol­ecules highlighting dominant short inter­atomic C—H/H—C and C—H⋯π/π⋯H—C inter­actions for the (*a*) O1-containing mol­ecule and (*b*) the O4-mol­ecule.

On the Hirshfeld surfaces mapped over the electrostatic potential for the independent mol­ecules of (I), Fig. 5 ▸, the donors and acceptors of inter­molecular inter­actions are represented with blue and red regions corresponding to positive and negative electrostatic potentials, respectively. The views of Hirshfeld surfaces about reference independent mol­ecules of (I) mapped within the shape-index property, Fig. 6 ▸, highlight the short inter­atomic C⋯H/H⋯C and C—H⋯π/π⋯H—C contacts operating in the crystal.

**Figure 7:**
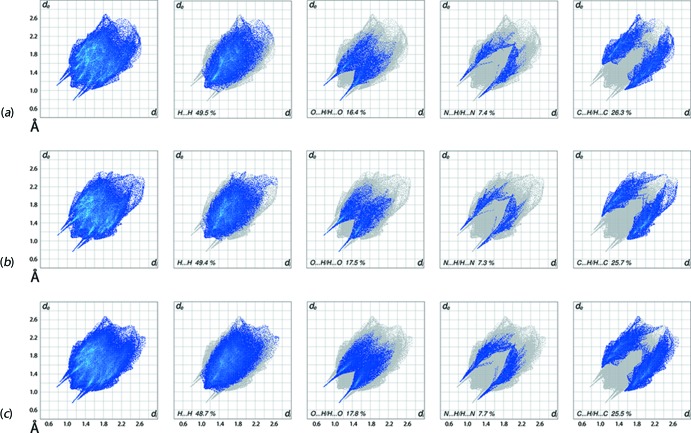
The full two-dimensional fingerprint plot and those delineated into H⋯H, O⋯H/H⋯O, N⋯H/H⋯N and C⋯H/H⋯C contacts for the (*a*) O1-containing mol­ecule, (*b*) the O4-mol­ecule and (*c*) (I).

It is clear from the overall two-dimensional fingerprint plots for each independent mol­ecule and for the entire asymmetric unit of (I) shown in Fig. 7 ▸ that the individual mol­ecules have common features in their inter­molecular O—H⋯N, N—H⋯O and C—H⋯π inter­actions. The small differences in the distribution of points in the fingerprint plots delineated into H⋯H, O⋯H/H⋯O, N⋯H/H⋯N and C⋯H/H⋯C contacts (McKinnon *et al.*, 2007 ▸) in Fig. 7 ▸, are ascribed to the commented upon (§3) conformational differences, *i.e*. the twisting of the meth­oxy substituents on the respective benzene rings and the inclination of these benzene rings with respect to the ethane bridges.

The fingerprint plot delineated into H⋯H contacts for mol­ecules *A* and *B* have almost the same percentage contribution to their respective Hirshfeld surfaces, Table 3 ▸, and the distinct distributions in the upper regions of the plots are due to the contributions from hydrogen atoms of their respective disubstituted benzene rings to the surfaces of mol­ecules *A* and *B*. The single short peaks at *d*_e_ + *d*_i_ ∼ 2.1 Å in the delineated plots for both the mol­ecules indicate the involvement of hydrogen atoms of both in short inter­atomic H⋯H contacts, Table 2 ▸. The inter­molecular N—H⋯O and O—H⋯N hydrogen bonds in the crystal are characterized as the pairs of spikes with their tips at *d*_e_ + *d*_i_ ∼ 2.0 Å (inner region) and at ∼ 2.2 Å (outer region) in the fingerprint plots delineated into O⋯H/H⋯O and N⋯H/H⋯N contacts, respectively. The forceps-like distribution of points linked with the donor spike for mol­ecule *A* and the acceptor spike for mol­ecule *B* at *d*_e_ + *d*_i_ ∼ 2.5 Å in the fingerprint plots delineated into O⋯H/H⋯O contacts are due to weak inter­molecular C—H⋯O inter­actions and the short inter­atomic contacts summarized in Table 2 ▸. The asymmetric forceps-like distribution of points with the tips at *d*_e_ + *d*_i_ ∼ 2.6 Å in the acceptor and donor regions of fingerprint plots delineated into C⋯H/H⋯C contacts for mol­ecules *A* and *B*, respectively, represent the involvement of these atoms in the short inter­atomic C⋯H/H⋯C contacts, Table 2 ▸, whereas the inter­molecular C—H⋯π inter­actions are viewed as the forceps-like tips at *d*_e_ + *d*_i_ ∼ 2.7 Å in the donor and acceptor regions of mol­ecules *A* and *B*, respectively. The other C⋯O/O⋯C, O⋯O and C⋯C inter­atomic contacts summarized in Table 3 ▸, having only small contributions to the Hirshfeld surface, have negligible directional impact on the mol­ecular packing.

## Database survey   

There are no direct precedents for the structure of (I) in the crystallographic literature (Groom *et al.*, 2016 ▸). However, there are several precedents for the phenyl­semicarbazone residue with the imine-carbon atom incorporated within an all-carbon ring (Groth, 1980 ▸; Hoek van den *et al.*, 1980 ▸), as exemplified in the cyclo­decane derivative (II) (Groth, 1980 ▸; Hoek van den *et al.*, 1980 ▸), see Scheme 2 for the chemical diagram of (II). More exotic derivatives with cyclic residues at both ends of the semicarbazone core are also known (Behenna *et al.*, 2011 ▸; Ma *et al.*, 2014 ▸), as exemplified by (III) (Ma *et al.*, 2014 ▸), Scheme 2.
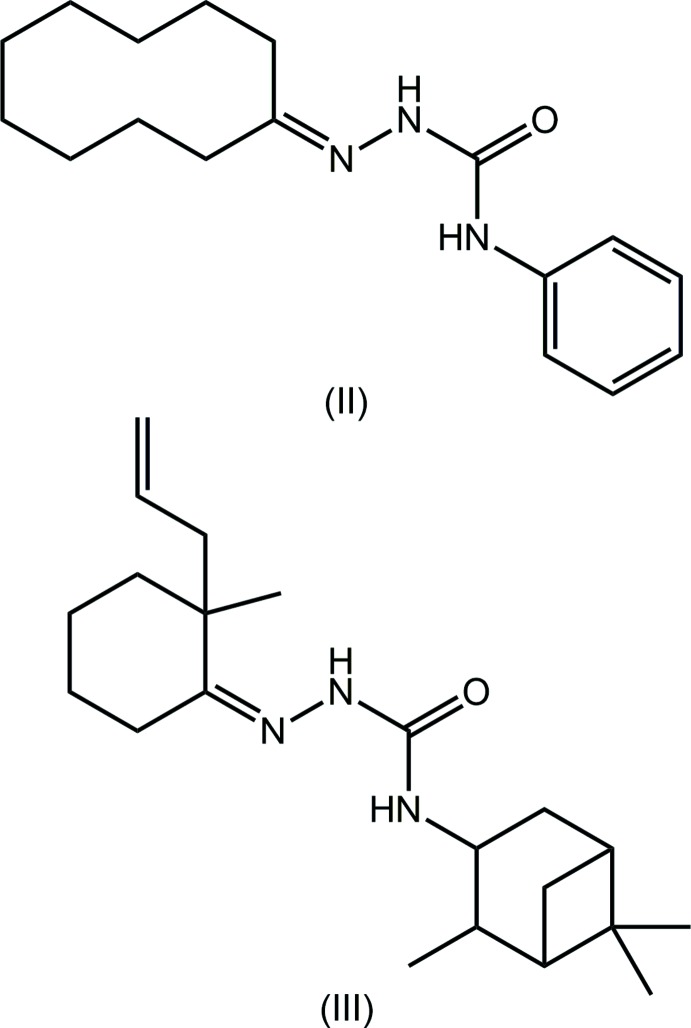


## Synthesis and crystallization   

Analytical grade reagents were used as procured without further purification. 4-Phenyl­semicarbazide (1.51 g, 0.01 mol) and vanillylacetone (1.94 g, 0.01 mol) were dissolved sep­arately in hot absolute ethanol (30 ml) and mixed with stirring. The reaction mixture was heated and stirred for 20 min., then stirred for another 30 min. at room temperature. The resulting white precipitate was filtered off, washed with cold absolute ethanol and dried *in vacuo*; yield: 75%. Light-yellow prisms of (I) were grown at room temperature from slow evaporation of mixed solvents of ethanol and aceto­nitrile (1:1; *v*/*v* 20 ml). IR (cm^−1^): 3201 ν(N—H), 1670 ν(C=N), 1213 ν(C—N), 1026 ν(C=O). MS *m*/*z*: 327.25 [*M*+1]^+^

## Refinement   

Crystal data, data collection and structure refinement details are summarized in Table 4 ▸. The carbon-bound H atoms were placed in calculated positions (C—H = 0.95–0.99 Å) and were included in the refinement in the riding-model approximation, with *U*_iso_(H) set to 1.2–1.5*U*_eq_(C). The oxygen- and nitro­gen-bound H atoms were located in a difference-Fourier map but were refined with distance restraints of O—H = 0.84±0.01 Å and N—H = 0.88±0.01 Å, and with *U*_iso_(H) set to 1.5*U*_eq_(O) and 1.2*U*_eq_(N), respectively. The maximum and minimum residual electron density peaks of 0.60 and 0.26 e Å^−3^, respectively, were located 0.95 and 0.75 Å from atoms H10*A* and H36*A*, respectively.

## Supplementary Material

Crystal structure: contains datablock(s) I, global. DOI: 10.1107/S2056989017017273/hb7720sup1.cif

Structure factors: contains datablock(s) I. DOI: 10.1107/S2056989017017273/hb7720Isup2.hkl

Supporting information file. DOI: 10.1107/S2056989017017273/hb7720Isup3.cml

CCDC reference: 926756

Additional supporting information:  crystallographic information; 3D view; checkCIF report

## Figures and Tables

**Table 1:** Hydrogen-bond geometry (Å, °) *Cg*1, *Cg*2 and *Cg*3 are the centroids of the C2–C7, C29–C34 and C20–C25 rings, respectively.

*D*—H⋯*A*	*D*—H	H⋯*A*	*D*⋯*A*	*D*—H⋯*A*
N1—H1*N*⋯N3	0.86 (2)	2.18 (2)	2.635 (2)	113 (2)
N4—H4*N*⋯N6	0.86 (2)	2.23 (2)	2.637 (2)	109 (1)
O3—H3*O*⋯O2	0.84 (2)	2.29 (3)	2.660 (2)	107 (2)
O6—H6*O*⋯O5	0.84 (2)	2.28 (2)	2.663 (2)	108 (2)
O3—H3*O*⋯N6^i^	0.84 (2)	2.19 (2)	2.994 (2)	161 (2)
O6—H6*O*⋯N3^ii^	0.84 (2)	2.22 (2)	3.013 (2)	157 (2)
N2—H2*N*⋯O4^iii^	0.88 (2)	2.01 (2)	2.873 (2)	170 (2)
N4—H4*N*⋯O2^i^	0.86 (2)	2.54 (2)	3.390 (2)	167 (2)
N5—H5*N*⋯O1^iv^	0.88 (2)	2.04 (2)	2.900 (2)	169 (2)
C33—H33⋯O6^v^	0.95	2.54	3.212 (2)	128
C15—H15⋯O3^vi^	0.95	2.63	3.166 (2)	113
C33—H33⋯O6^i^	0.95	2.54	3.212 (2)	128
C10—H10*A*⋯*Cg*1^vii^	0.99	2.80	3.774 (2)	168
C18—H18*A*⋯*Cg*2^ii^	0.98	2.66	3.603 (4)	161
C28—H28*B*⋯*Cg*3^viii^	0.99	2.75	3.720 (2)	166

Symmetry codes: (i) 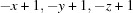; (ii) 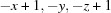; (iii) 
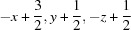
; (iv) 
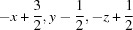
; (v) 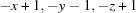; (vi) 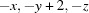; (vii) 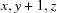; (viii) 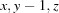.

**Table 2:** Summary of short inter­atomic contacts (Å) in (I)

Contact	Distance	Symmetry operation
H3⋯H21	2.16	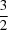 − *x*, − 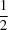 + *y*, 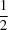 − *z*
H28*A*⋯H35*A*	2.24	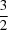 − *x*, − 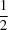 + *y*, 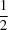 − *z*
O1⋯H22	2.50	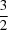 − *x*, − 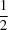 + *y*, 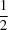 − *z*
O2⋯H27*B*	2.56	1 − *x*, 1 − *y*, 1 − *z*
O3⋯H15	2.63	−*x*, 2 − *y*, 1 − *z*
O3⋯C15	3.166 (2)	−*x*, 2 − *y*, 1 − *z*
O3⋯H23	2.58	1 − *x*, 2 − *y*, 1 − *z*
O4⋯H16	2.58	1 + *x*, −1 + *y*, *z*
O5⋯H9*A*	2.46	1 − *x*, −*y*, 1 − *z*
C6⋯H10*A*	2.63	*x*, −1 + *y*, *z*
C15⋯H36*C*	2.55	1 − *x*, 1 − *y*, 1 − *z*
C16⋯H36*C*	2.80	1 − *x*, 1 − *y*, 1 − *z*
C24⋯H28*B*	2.63	*x*, 1 + *y*, *z*
C32⋯H18*A*	2.76	1 − *x*, − *y*, 1 − *z*
C33⋯H18*A*	2.62	1 − *x*, − *y*, 1 − *z*
C34⋯H18*A*	2.78	1 − *x*, − *y*, 1 − *z*
C35⋯H18*B*	2.71	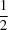 + *x*, 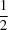 − *y*, − 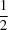 + *z*
C36⋯H9*A*	2.80	1 − *x*, − *y*, 1 − *z*
C6⋯C6	3.210 (3)	1 − *x*, − *y*, 1 − *z*
C24⋯C24	3.300 (3)	2 − *x*, 1 − *y*, 1 − *z*

**Table 3:** Percentage contributions of inter­atomic contacts to the Hirshfeld surface for the O1-mol­ecule, the O4-mol­ecule and for overall (I)

Contact	Percentage contribution		
	O1-mol­ecule	O4-mol­ecule	(I)
H⋯H	49.5	49.4	48.7
O⋯H/H⋯O	16.4	17.5	17.8
N⋯H/H⋯N	7.4	7.3	7.7
C⋯H/H⋯C	26.3	25.7	25.5
C⋯C	0.1	0.1	0.1
O⋯O	0.2	0.0	0.1
C⋯O/O⋯C	0.1	0.0	0.1

**Table 4:** Experimental details

Crystal data	
Chemical formula	C_18_H_21_N_3_O_3_
*M*_r_	327.37
Crystal system, space group	Monoclinic, *P*2_1_/*n*
Temperature (K)	100
*a*, *b*, *c* (Å)	16.5464 (4), 9.2184 (2), 22.3975 (4)
β (°)	100.494 (2)
*V* (Å^3^)	3359.18 (13)
*Z*	8
Radiation type	Cu *K*α
μ (mm^−1^)	0.73
Crystal size (mm)	0.25 × 0.16 × 0.06

Data collection	
Diffractometer	Oxford Diffraction Xcaliber Eos Gemini
Absorption correction	Multi-scan (*CrysAlis PRO*; Agilent, 2011 ▸)
*T*_min_, *T*_max_	0.917, 1.000
No. of measured, independent and observed [*I* > 2σ(*I*)] reflections	23815, 6481, 5581
*R*_int_	0.019
(sin θ/λ)_max_ (Å^−1^)	0.615

Refinement	
*R*[*F* ^2^ > 2σ(*F* ^2^)], *wR*(*F* ^2^), *S*	0.059, 0.180, 1.05
No. of reflections	6481
No. of parameters	455
No. of restraints	6
H-atom treatment	H atoms treated by a mixture of independent and constrained refinement
Δρ_max_, Δρ_min_ (e Å^−3^)	0.60, −0.26

Computer programs: *CrysAlis PRO* (Agilent, 2011 ▸), *SHELXS97* (Sheldrick, 2008 ▸), *SHELXL2014* (Sheldrick, 2015 ▸), *ORTEP-3 for Windows* (Farrugia, 2012 ▸), *DIAMOND* (Brandenburg, 2006 ▸) and *publCIF* (Westrip, 2010 ▸).

## supplementary crystallographic information

### Crystal data

**Table:**

C_18_H_21_N_3_O_3_	*F*(000) = 1392
*M**_r_* = 327.37	*D*_x_ = 1.295 Mg m^−^^3^
Monoclinic, *P*2_1_/*n*	Cu *K*α radiation, λ = 1.5418 Å
*a* = 16.5464 (4) Å	Cell parameters from 9245 reflections
*b* = 9.2184 (2) Å	θ = 3.7–71.3°
*c* = 22.3975 (4) Å	µ = 0.73 mm^−^^1^
β = 100.494 (2)°	*T* = 100 K
*V* = 3359.18 (13) Å^3^	Prism (cut), light-yellow
*Z* = 8	0.25 × 0.16 × 0.06 mm

### Data collection

**Table:**

Oxford Diffraction Xcaliber Eos Gemini diffractometer	6481 independent reflections
Radiation source: fine-focus sealed tube	5581 reflections with *I* > 2σ(*I*)
Graphite monochromator	*R*_int_ = 0.019
Detector resolution: 16.1952 pixels mm^-1^	θ_max_ = 71.4°, θ_min_ = 3.7°
ω scans	*h* = −20→20
Absorption correction: multi-scan (CrysAlis PRO; Agilent, 2011)	*k* = −11→11
*T*_min_ = 0.917, *T*_max_ = 1.000	*l* = −26→27
23815 measured reflections	

### Refinement

**Table:**

Refinement on *F*^2^	6 restraints
Least-squares matrix: full	Hydrogen site location: mixed
*R*[*F*^2^ > 2σ(*F*^2^)] = 0.059	H atoms treated by a mixture of independent and constrained refinement
*wR*(*F*^2^) = 0.180	*w* = 1/[σ^2^(*F*_o_^2^) + (0.1131*P*)^2^ + 1.5134*P*] where *P* = (*F*_o_^2^ + 2*F*_c_^2^)/3
*S* = 1.05	(Δ/σ)_max_ < 0.001
6481 reflections	Δρ_max_ = 0.60 e Å^−^^3^
455 parameters	Δρ_min_ = −0.26 e Å^−^^3^

### Special details

**Table:**

Geometry. All esds (except the esd in the dihedral angle between two l.s. planes) are estimated using the full covariance matrix. The cell esds are taken into account individually in the estimation of esds in distances, angles and torsion angles; correlations between esds in cell parameters are only used when they are defined by crystal symmetry. An approximate (isotropic) treatment of cell esds is used for estimating esds involving l.s. planes.

### Fractional atomic coordinates and isotropic or equivalent isotropic displacement parameters (Å^2^)

**Table:**

	*x*	*y*	*z*	*U*_iso_*/*U*_eq_	
O1	0.50355 (7)	0.34300 (14)	0.28183 (6)	0.0292 (3)	
O2	0.22012 (9)	0.87251 (16)	0.56672 (6)	0.0386 (4)	
O3	0.09139 (8)	1.04987 (16)	0.54412 (6)	0.0350 (3)	
H3O	0.1190 (15)	1.034 (3)	0.5787 (7)	0.053*	
N1	0.40785 (9)	0.29389 (16)	0.34177 (6)	0.0253 (3)	
H1N	0.3694 (10)	0.337 (2)	0.3564 (9)	0.030*	
N2	0.40724 (9)	0.51144 (16)	0.29066 (7)	0.0256 (3)	
H2N	0.4313 (12)	0.5727 (19)	0.2696 (8)	0.031*	
N3	0.34334 (9)	0.55389 (17)	0.31934 (7)	0.0284 (3)	
C1	0.44364 (10)	0.37902 (19)	0.30419 (7)	0.0238 (3)	
C2	0.43316 (10)	0.15426 (19)	0.36316 (8)	0.0259 (4)	
C3	0.47454 (13)	0.0593 (2)	0.33074 (9)	0.0360 (4)	
H3	0.4884	0.0887	0.2932	0.043*	
C4	0.49550 (13)	−0.0785 (2)	0.35355 (10)	0.0407 (5)	
H4	0.5243	−0.1425	0.3316	0.049*	
C5	0.47517 (13)	−0.1240 (2)	0.40757 (10)	0.0419 (5)	
H5	0.4894	−0.2186	0.4227	0.050*	
C6	0.43353 (14)	−0.0291 (2)	0.43949 (10)	0.0442 (5)	
H6	0.4192	−0.0594	0.4768	0.053*	
C7	0.41258 (13)	0.1094 (2)	0.41765 (9)	0.0368 (4)	
H7	0.3842	0.1734	0.4400	0.044*	
C8	0.29887 (11)	0.6607 (2)	0.29621 (8)	0.0297 (4)	
C9	0.23057 (11)	0.7061 (2)	0.32858 (9)	0.0326 (4)	
H9A	0.2323	0.6455	0.3653	0.039*	
H9B	0.1769	0.6900	0.3016	0.039*	
C10	0.23837 (13)	0.8663 (2)	0.34707 (9)	0.0355 (4)	
H10A	0.2974	0.8905	0.3592	0.043*	
H10B	0.2158	0.9266	0.3113	0.043*	
C11	0.19461 (12)	0.9052 (2)	0.39874 (8)	0.0329 (4)	
C12	0.22808 (13)	0.8620 (2)	0.45796 (9)	0.0367 (4)	
H12	0.2757	0.8024	0.4650	0.044*	
C13	0.19215 (12)	0.9056 (2)	0.50660 (8)	0.0307 (4)	
C14	0.12349 (11)	0.99627 (19)	0.49664 (8)	0.0261 (4)	
C15	0.08828 (11)	1.0335 (2)	0.43795 (9)	0.0302 (4)	
H15	0.0400	1.0915	0.4307	0.036*	
C16	0.12319 (11)	0.9866 (2)	0.38942 (8)	0.0322 (4)	
H16	0.0976	1.0108	0.3492	0.039*	
C17	0.31178 (11)	0.7458 (2)	0.24149 (8)	0.0329 (4)	
H17A	0.3595	0.8096	0.2526	0.049*	
H17B	0.2628	0.8043	0.2266	0.049*	
H17C	0.3214	0.6788	0.2095	0.049*	
C18	0.30011 (18)	0.8105 (4)	0.58127 (11)	0.0742 (10)	
H18A	0.2995	0.7133	0.5635	0.111*	
H18B	0.3167	0.8039	0.6255	0.111*	
H18C	0.3393	0.8718	0.5648	0.111*	
O4	0.99721 (7)	0.19097 (14)	0.27509 (6)	0.0296 (3)	
O5	0.74121 (9)	−0.41262 (16)	0.57259 (6)	0.0389 (3)	
O6	0.59792 (8)	−0.55085 (16)	0.54521 (6)	0.0355 (3)	
H6O	0.6277 (14)	−0.547 (3)	0.5798 (7)	0.053*	
N4	0.90978 (8)	0.23778 (16)	0.34148 (6)	0.0245 (3)	
H4N	0.8743 (10)	0.198 (2)	0.3602 (8)	0.029*	
N5	0.90487 (9)	0.02044 (16)	0.28954 (7)	0.0263 (3)	
H5N	0.9267 (12)	−0.040 (2)	0.2668 (8)	0.032*	
N6	0.84546 (9)	−0.02354 (17)	0.32229 (7)	0.0288 (3)	
C19	0.94100 (10)	0.15314 (18)	0.30127 (7)	0.0230 (3)	
C20	0.93614 (10)	0.37987 (19)	0.35945 (8)	0.0251 (4)	
C21	0.96816 (12)	0.4747 (2)	0.32082 (8)	0.0309 (4)	
H21	0.9735	0.4440	0.2812	0.037*	
C22	0.99218 (12)	0.6136 (2)	0.34046 (9)	0.0350 (4)	
H22	1.0146	0.6768	0.3142	0.042*	
C23	0.98404 (12)	0.6617 (2)	0.39743 (9)	0.0367 (4)	
H23	1.0006	0.7571	0.4104	0.044*	
C24	0.95139 (13)	0.5686 (2)	0.43530 (9)	0.0384 (5)	
H24	0.9451	0.6008	0.4745	0.046*	
C25	0.92765 (12)	0.4282 (2)	0.41667 (8)	0.0331 (4)	
H25	0.9056	0.3653	0.4432	0.040*	
C26	0.79860 (11)	−0.1278 (2)	0.30028 (8)	0.0292 (4)	
C27	0.73558 (11)	−0.1766 (2)	0.33697 (9)	0.0330 (4)	
H27A	0.6797	−0.1593	0.3134	0.040*	
H27B	0.7417	−0.1189	0.3748	0.040*	
C28	0.74571 (13)	−0.3380 (2)	0.35290 (9)	0.0364 (4)	
H28A	0.7238	−0.3958	0.3163	0.044*	
H28B	0.8051	−0.3600	0.3645	0.044*	
C29	0.70304 (12)	−0.3848 (2)	0.40393 (9)	0.0327 (4)	
C30	0.74324 (12)	−0.3697 (2)	0.46427 (9)	0.0342 (4)	
H30	0.7957	−0.3245	0.4729	0.041*	
C31	0.70709 (12)	−0.4203 (2)	0.51172 (8)	0.0307 (4)	
C32	0.63080 (11)	−0.49006 (19)	0.49940 (8)	0.0265 (4)	
C33	0.58946 (11)	−0.5002 (2)	0.44001 (9)	0.0320 (4)	
H33	0.5367	−0.5443	0.4314	0.038*	
C34	0.62477 (12)	−0.4461 (2)	0.39286 (8)	0.0326 (4)	
H34	0.5951	−0.4510	0.3524	0.039*	
C35	0.80405 (11)	−0.2075 (2)	0.24253 (8)	0.0329 (4)	
H35A	0.8160	−0.1383	0.2120	0.049*	
H35B	0.7516	−0.2560	0.2274	0.049*	
H35C	0.8481	−0.2799	0.2506	0.049*	
C36	0.82787 (14)	−0.3844 (3)	0.58750 (10)	0.0481 (6)	
H36A	0.8572	−0.4519	0.5652	0.072*	
H36B	0.8468	−0.3976	0.6312	0.072*	
H36C	0.8388	−0.2845	0.5762	0.072*	

### Atomic displacement parameters (Å^2^)

**Table:**

	*U*^11^	*U*^22^	*U*^33^	*U*^12^	*U*^13^	*U*^23^
O1	0.0302 (6)	0.0278 (6)	0.0345 (7)	0.0047 (5)	0.0187 (5)	0.0035 (5)
O2	0.0518 (8)	0.0411 (8)	0.0266 (7)	0.0180 (6)	0.0167 (6)	0.0056 (5)
O3	0.0351 (7)	0.0431 (8)	0.0304 (7)	0.0075 (6)	0.0154 (5)	−0.0057 (6)
N1	0.0276 (7)	0.0269 (7)	0.0248 (7)	0.0014 (6)	0.0136 (6)	−0.0006 (6)
N2	0.0257 (7)	0.0268 (7)	0.0281 (7)	0.0019 (6)	0.0153 (6)	0.0016 (6)
N3	0.0294 (7)	0.0313 (8)	0.0282 (8)	0.0033 (6)	0.0152 (6)	−0.0005 (6)
C1	0.0241 (8)	0.0256 (8)	0.0227 (8)	−0.0003 (6)	0.0073 (6)	−0.0015 (6)
C2	0.0271 (8)	0.0268 (9)	0.0245 (8)	−0.0036 (7)	0.0064 (6)	0.0007 (7)
C3	0.0475 (11)	0.0307 (10)	0.0352 (10)	0.0047 (8)	0.0222 (9)	0.0055 (8)
C4	0.0455 (11)	0.0308 (10)	0.0500 (12)	0.0066 (8)	0.0196 (9)	0.0071 (9)
C5	0.0435 (11)	0.0326 (10)	0.0498 (12)	−0.0010 (8)	0.0088 (9)	0.0140 (9)
C6	0.0571 (13)	0.0425 (12)	0.0363 (11)	−0.0079 (10)	0.0172 (9)	0.0121 (9)
C7	0.0463 (11)	0.0359 (10)	0.0320 (10)	−0.0043 (8)	0.0176 (8)	0.0001 (8)
C8	0.0296 (9)	0.0336 (9)	0.0277 (9)	0.0013 (7)	0.0097 (7)	−0.0028 (7)
C9	0.0283 (9)	0.0367 (10)	0.0353 (10)	0.0005 (7)	0.0123 (7)	−0.0034 (8)
C10	0.0449 (11)	0.0343 (10)	0.0314 (10)	0.0030 (8)	0.0179 (8)	0.0023 (8)
C11	0.0419 (10)	0.0292 (9)	0.0307 (9)	0.0023 (8)	0.0149 (8)	−0.0010 (7)
C12	0.0444 (11)	0.0351 (10)	0.0340 (10)	0.0142 (8)	0.0164 (8)	0.0035 (8)
C13	0.0395 (10)	0.0289 (9)	0.0265 (9)	0.0058 (7)	0.0134 (7)	0.0019 (7)
C14	0.0292 (9)	0.0239 (8)	0.0291 (9)	−0.0029 (6)	0.0163 (7)	−0.0021 (6)
C15	0.0246 (8)	0.0327 (9)	0.0354 (10)	0.0004 (7)	0.0110 (7)	0.0012 (7)
C16	0.0333 (9)	0.0384 (10)	0.0262 (9)	0.0011 (8)	0.0087 (7)	0.0005 (7)
C17	0.0342 (9)	0.0383 (10)	0.0289 (9)	0.0117 (8)	0.0133 (7)	0.0041 (7)
C18	0.0821 (19)	0.111 (3)	0.0325 (11)	0.0655 (19)	0.0182 (12)	0.0211 (13)
O4	0.0312 (6)	0.0265 (6)	0.0362 (7)	−0.0032 (5)	0.0200 (5)	−0.0031 (5)
O5	0.0459 (8)	0.0460 (8)	0.0266 (7)	−0.0145 (6)	0.0118 (6)	−0.0018 (6)
O6	0.0317 (7)	0.0471 (8)	0.0312 (7)	−0.0050 (6)	0.0152 (5)	0.0059 (6)
N4	0.0245 (7)	0.0272 (7)	0.0244 (7)	−0.0009 (5)	0.0116 (5)	0.0008 (5)
N5	0.0271 (7)	0.0279 (8)	0.0279 (7)	−0.0029 (6)	0.0159 (6)	−0.0030 (6)
N6	0.0296 (8)	0.0313 (8)	0.0296 (8)	−0.0042 (6)	0.0166 (6)	−0.0001 (6)
C19	0.0223 (8)	0.0250 (8)	0.0230 (8)	0.0008 (6)	0.0075 (6)	0.0015 (6)
C20	0.0223 (8)	0.0286 (9)	0.0249 (8)	0.0030 (6)	0.0057 (6)	−0.0027 (7)
C21	0.0371 (10)	0.0287 (9)	0.0297 (9)	−0.0013 (7)	0.0140 (7)	−0.0046 (7)
C22	0.0376 (10)	0.0287 (10)	0.0421 (11)	−0.0032 (8)	0.0162 (8)	−0.0037 (8)
C23	0.0379 (10)	0.0301 (10)	0.0431 (11)	0.0004 (8)	0.0104 (8)	−0.0121 (8)
C24	0.0458 (11)	0.0394 (11)	0.0313 (10)	0.0059 (9)	0.0103 (8)	−0.0108 (8)
C25	0.0384 (10)	0.0346 (10)	0.0286 (9)	0.0039 (8)	0.0125 (8)	−0.0005 (7)
C26	0.0275 (9)	0.0306 (9)	0.0311 (9)	−0.0004 (7)	0.0098 (7)	0.0036 (7)
C27	0.0293 (9)	0.0332 (10)	0.0395 (10)	−0.0004 (7)	0.0144 (8)	0.0038 (8)
C28	0.0450 (11)	0.0340 (10)	0.0345 (10)	0.0026 (8)	0.0186 (8)	0.0026 (8)
C29	0.0403 (10)	0.0281 (9)	0.0326 (10)	0.0010 (7)	0.0143 (8)	0.0029 (7)
C30	0.0373 (10)	0.0318 (10)	0.0361 (10)	−0.0073 (8)	0.0131 (8)	−0.0007 (8)
C31	0.0375 (10)	0.0296 (9)	0.0270 (9)	−0.0041 (7)	0.0111 (7)	−0.0026 (7)
C32	0.0288 (9)	0.0249 (8)	0.0299 (9)	0.0034 (7)	0.0165 (7)	0.0012 (7)
C33	0.0241 (8)	0.0377 (10)	0.0357 (10)	0.0035 (7)	0.0095 (7)	0.0007 (8)
C34	0.0341 (9)	0.0365 (10)	0.0280 (9)	0.0043 (8)	0.0077 (7)	0.0021 (7)
C35	0.0330 (9)	0.0400 (11)	0.0267 (9)	−0.0114 (8)	0.0076 (7)	−0.0018 (7)
C36	0.0474 (12)	0.0608 (15)	0.0358 (11)	−0.0236 (11)	0.0064 (9)	−0.0054 (10)

### Geometric parameters (Å, º)

**Table:**

O1—C1	1.235 (2)	O4—C19	1.237 (2)
O2—C13	1.376 (2)	O5—C31	1.379 (2)
O2—C18	1.424 (3)	O5—C36	1.436 (2)
O3—C14	1.365 (2)	O6—C32	1.366 (2)
O3—H3O	0.838 (10)	O6—H6O	0.840 (10)
N1—C1	1.363 (2)	N4—C19	1.362 (2)
N1—C2	1.410 (2)	N4—C20	1.416 (2)
N1—H1N	0.862 (9)	N4—H4N	0.865 (9)
N2—C1	1.371 (2)	N5—C19	1.366 (2)
N2—N3	1.3895 (19)	N5—N6	1.3899 (19)
N2—H2N	0.876 (9)	N5—H5N	0.878 (9)
N3—C8	1.281 (2)	N6—C26	1.276 (2)
C2—C7	1.388 (2)	C20—C25	1.388 (2)
C2—C3	1.394 (3)	C20—C21	1.400 (3)
C3—C4	1.390 (3)	C21—C22	1.389 (3)
C3—H3	0.9500	C21—H21	0.9500
C4—C5	1.379 (3)	C22—C23	1.380 (3)
C4—H4	0.9500	C22—H22	0.9500
C5—C6	1.389 (3)	C23—C24	1.384 (3)
C5—H5	0.9500	C23—H23	0.9500
C6—C7	1.388 (3)	C24—C25	1.394 (3)
C6—H6	0.9500	C24—H24	0.9500
C7—H7	0.9500	C25—H25	0.9500
C8—C17	1.503 (2)	C26—C35	1.504 (3)
C8—C9	1.509 (2)	C26—C27	1.509 (2)
C9—C10	1.533 (3)	C27—C28	1.532 (3)
C9—H9A	0.9900	C27—H27A	0.9900
C9—H9B	0.9900	C27—H27B	0.9900
C10—C11	1.516 (2)	C28—C29	1.512 (2)
C10—H10A	0.9900	C28—H28A	0.9900
C10—H10B	0.9900	C28—H28B	0.9900
C11—C16	1.383 (3)	C29—C34	1.393 (3)
C11—C12	1.399 (3)	C29—C30	1.400 (3)
C12—C13	1.392 (2)	C30—C31	1.392 (3)
C12—H12	0.9500	C30—H30	0.9500
C13—C14	1.395 (3)	C31—C32	1.399 (3)
C14—C15	1.381 (3)	C32—C33	1.384 (3)
C15—C16	1.389 (3)	C33—C34	1.389 (3)
C15—H15	0.9500	C33—H33	0.9500
C16—H16	0.9500	C34—H34	0.9500
C17—H17A	0.9800	C35—H35A	0.9800
C17—H17B	0.9800	C35—H35B	0.9800
C17—H17C	0.9800	C35—H35C	0.9800
C18—H18A	0.9800	C36—H36A	0.9800
C18—H18B	0.9800	C36—H36B	0.9800
C18—H18C	0.9800	C36—H36C	0.9800
			
C13—O2—C18	116.47 (15)	C31—O5—C36	116.80 (15)
C14—O3—H3O	115 (2)	C32—O6—H6O	115 (2)
C1—N1—C2	126.87 (14)	C19—N4—C20	125.70 (14)
C1—N1—H1N	113.9 (15)	C19—N4—H4N	116.7 (15)
C2—N1—H1N	118.8 (15)	C20—N4—H4N	117.3 (14)
C1—N2—N3	119.30 (14)	C19—N5—N6	119.11 (14)
C1—N2—H2N	117.9 (14)	C19—N5—H5N	118.1 (14)
N3—N2—H2N	121.7 (14)	N6—N5—H5N	121.7 (15)
C8—N3—N2	117.32 (15)	C26—N6—N5	117.01 (15)
O1—C1—N1	124.55 (16)	O4—C19—N4	124.09 (15)
O1—C1—N2	120.18 (15)	O4—C19—N5	120.13 (15)
N1—C1—N2	115.26 (14)	N4—C19—N5	115.77 (14)
C7—C2—C3	119.56 (17)	C25—C20—C21	118.93 (17)
C7—C2—N1	117.72 (16)	C25—C20—N4	118.80 (16)
C3—C2—N1	122.67 (15)	C21—C20—N4	122.24 (15)
C4—C3—C2	119.74 (17)	C22—C21—C20	119.91 (17)
C4—C3—H3	120.1	C22—C21—H21	120.0
C2—C3—H3	120.1	C20—C21—H21	120.0
C5—C4—C3	121.05 (19)	C23—C22—C21	121.23 (18)
C5—C4—H4	119.5	C23—C22—H22	119.4
C3—C4—H4	119.5	C21—C22—H22	119.4
C4—C5—C6	118.92 (19)	C22—C23—C24	118.82 (18)
C4—C5—H5	120.5	C22—C23—H23	120.6
C6—C5—H5	120.5	C24—C23—H23	120.6
C7—C6—C5	120.86 (19)	C23—C24—C25	120.83 (18)
C7—C6—H6	119.6	C23—C24—H24	119.6
C5—C6—H6	119.6	C25—C24—H24	119.6
C6—C7—C2	119.87 (19)	C20—C25—C24	120.26 (18)
C6—C7—H7	120.1	C20—C25—H25	119.9
C2—C7—H7	120.1	C24—C25—H25	119.9
N3—C8—C17	125.06 (16)	N6—C26—C35	124.91 (16)
N3—C8—C9	116.39 (16)	N6—C26—C27	116.46 (16)
C17—C8—C9	118.50 (16)	C35—C26—C27	118.58 (16)
C8—C9—C10	111.32 (16)	C26—C27—C28	111.04 (15)
C8—C9—H9A	109.4	C26—C27—H27A	109.4
C10—C9—H9A	109.4	C28—C27—H27A	109.4
C8—C9—H9B	109.4	C26—C27—H27B	109.4
C10—C9—H9B	109.4	C28—C27—H27B	109.4
H9A—C9—H9B	108.0	H27A—C27—H27B	108.0
C11—C10—C9	113.94 (16)	C29—C28—C27	114.03 (16)
C11—C10—H10A	108.8	C29—C28—H28A	108.7
C9—C10—H10A	108.8	C27—C28—H28A	108.7
C11—C10—H10B	108.8	C29—C28—H28B	108.7
C9—C10—H10B	108.8	C27—C28—H28B	108.7
H10A—C10—H10B	107.7	H28A—C28—H28B	107.6
C16—C11—C12	118.52 (17)	C34—C29—C30	118.35 (17)
C16—C11—C10	121.80 (17)	C34—C29—C28	121.90 (18)
C12—C11—C10	119.66 (17)	C30—C29—C28	119.74 (17)
C13—C12—C11	120.53 (18)	C31—C30—C29	120.59 (17)
C13—C12—H12	119.7	C31—C30—H30	119.7
C11—C12—H12	119.7	C29—C30—H30	119.7
O2—C13—C12	125.91 (17)	O5—C31—C30	125.60 (17)
O2—C13—C14	114.09 (15)	O5—C31—C32	114.27 (16)
C12—C13—C14	119.94 (17)	C30—C31—C32	120.10 (17)
O3—C14—C15	119.68 (16)	O6—C32—C33	119.97 (16)
O3—C14—C13	120.88 (16)	O6—C32—C31	120.60 (16)
C15—C14—C13	119.44 (16)	C33—C32—C31	119.41 (16)
C14—C15—C16	120.25 (17)	C32—C33—C34	120.25 (17)
C14—C15—H15	119.9	C32—C33—H33	119.9
C16—C15—H15	119.9	C34—C33—H33	119.9
C11—C16—C15	121.08 (17)	C33—C34—C29	121.09 (17)
C11—C16—H16	119.5	C33—C34—H34	119.5
C15—C16—H16	119.5	C29—C34—H34	119.5
C8—C17—H17A	109.5	C26—C35—H35A	109.5
C8—C17—H17B	109.5	C26—C35—H35B	109.5
H17A—C17—H17B	109.5	H35A—C35—H35B	109.5
C8—C17—H17C	109.5	C26—C35—H35C	109.5
H17A—C17—H17C	109.5	H35A—C35—H35C	109.5
H17B—C17—H17C	109.5	H35B—C35—H35C	109.5
O2—C18—H18A	109.5	O5—C36—H36A	109.5
O2—C18—H18B	109.5	O5—C36—H36B	109.5
H18A—C18—H18B	109.5	H36A—C36—H36B	109.5
O2—C18—H18C	109.5	O5—C36—H36C	109.5
H18A—C18—H18C	109.5	H36A—C36—H36C	109.5
H18B—C18—H18C	109.5	H36B—C36—H36C	109.5
			
C1—N2—N3—C8	−164.49 (16)	C19—N5—N6—C26	162.35 (16)
C2—N1—C1—O1	2.0 (3)	C20—N4—C19—O4	0.0 (3)
C2—N1—C1—N2	−179.42 (15)	C20—N4—C19—N5	−178.54 (15)
N3—N2—C1—O1	−175.81 (15)	N6—N5—C19—O4	175.95 (15)
N3—N2—C1—N1	5.6 (2)	N6—N5—C19—N4	−5.5 (2)
C1—N1—C2—C7	154.49 (18)	C19—N4—C20—C25	−151.84 (17)
C1—N1—C2—C3	−28.1 (3)	C19—N4—C20—C21	30.1 (3)
C7—C2—C3—C4	−0.7 (3)	C25—C20—C21—C22	1.1 (3)
N1—C2—C3—C4	−178.00 (18)	N4—C20—C21—C22	179.19 (17)
C2—C3—C4—C5	0.8 (3)	C20—C21—C22—C23	−1.0 (3)
C3—C4—C5—C6	−0.4 (3)	C21—C22—C23—C24	0.1 (3)
C4—C5—C6—C7	0.0 (3)	C22—C23—C24—C25	0.5 (3)
C5—C6—C7—C2	0.2 (3)	C21—C20—C25—C24	−0.5 (3)
C3—C2—C7—C6	0.2 (3)	N4—C20—C25—C24	−178.64 (16)
N1—C2—C7—C6	177.68 (18)	C23—C24—C25—C20	−0.3 (3)
N2—N3—C8—C17	−1.6 (3)	N5—N6—C26—C35	1.1 (3)
N2—N3—C8—C9	−179.23 (15)	N5—N6—C26—C27	178.73 (15)
N3—C8—C9—C10	123.12 (19)	N6—C26—C27—C28	−122.47 (19)
C17—C8—C9—C10	−54.7 (2)	C35—C26—C27—C28	55.3 (2)
C8—C9—C10—C11	−157.88 (16)	C26—C27—C28—C29	162.93 (17)
C9—C10—C11—C16	−108.0 (2)	C27—C28—C29—C34	95.8 (2)
C9—C10—C11—C12	73.9 (2)	C27—C28—C29—C30	−85.6 (2)
C16—C11—C12—C13	−2.8 (3)	C34—C29—C30—C31	2.6 (3)
C10—C11—C12—C13	175.28 (18)	C28—C29—C30—C31	−176.05 (18)
C18—O2—C13—C12	11.7 (3)	C36—O5—C31—C30	−16.5 (3)
C18—O2—C13—C14	−165.4 (2)	C36—O5—C31—C32	161.41 (18)
C11—C12—C13—O2	−178.72 (19)	C29—C30—C31—O5	179.33 (18)
C11—C12—C13—C14	−1.7 (3)	C29—C30—C31—C32	1.5 (3)
O2—C13—C14—O3	2.7 (3)	O5—C31—C32—O6	−3.6 (3)
C12—C13—C14—O3	−174.61 (17)	C30—C31—C32—O6	174.50 (17)
O2—C13—C14—C15	−178.04 (16)	O5—C31—C32—C33	177.97 (16)
C12—C13—C14—C15	4.6 (3)	C30—C31—C32—C33	−4.0 (3)
O3—C14—C15—C16	176.25 (16)	O6—C32—C33—C34	−176.20 (17)
C13—C14—C15—C16	−3.0 (3)	C31—C32—C33—C34	2.3 (3)
C12—C11—C16—C15	4.5 (3)	C32—C33—C34—C29	1.9 (3)
C10—C11—C16—C15	−173.55 (18)	C30—C29—C34—C33	−4.4 (3)
C14—C15—C16—C11	−1.6 (3)	C28—C29—C34—C33	174.28 (18)

### Hydrogen-bond geometry (Å, º)

Cg1, Cg2 and Cg3 are the centroids of the C2–C7, C29–C34 and C20–C25 rings, respectively.

**Table:**

*D*—H···*A*	*D*—H	H···*A*	*D*···*A*	*D*—H···*A*
N1—H1*N*···N3	0.86 (2)	2.18 (2)	2.635 (2)	113 (2)
N4—H4*N*···N6	0.86 (2)	2.23 (2)	2.637 (2)	109 (1)
O3—H3*O*···O2	0.84 (2)	2.29 (3)	2.660 (2)	107 (2)
O6—H6*O*···O5	0.84 (2)	2.28 (2)	2.663 (2)	108 (2)
O3—H3*O*···N6^i^	0.84 (2)	2.19 (2)	2.994 (2)	161 (2)
O6—H6*O*···N3^ii^	0.84 (2)	2.22 (2)	3.013 (2)	157 (2)
N2—H2*N*···O4^iii^	0.88 (2)	2.01 (2)	2.873 (2)	170 (2)
N4—H4*N*···O2^i^	0.86 (2)	2.54 (2)	3.390 (2)	167 (2)
N5—H5*N*···O1^iv^	0.88 (2)	2.04 (2)	2.900 (2)	169 (2)
C33—H33···O6^v^	0.95	2.54	3.212 (2)	128
C15—H15···O3^vi^	0.95	2.63	3.166 (2)	113
C33—H33···O6^i^	0.95	2.54	3.212 (2)	128
C10—H10*A*···*Cg*1^vii^	0.99	2.80	3.774 (2)	168
C18—H18*A*···*Cg*2^ii^	0.98	2.66	3.603 (4)	161
C28—H28*B*···*Cg*3^viii^	0.99	2.75	3.720 (2)	166

Symmetry codes: (i) −*x*+1, −*y*+1, −*z*+1; (ii) −*x*+1, −*y*, −*z*+1; (iii) −*x*+3/2, *y*+1/2, −*z*+1/2; (iv) −*x*+3/2, *y*−1/2, −*z*+1/2; (v) −*x*+1, −*y*−1, −*z*+1; (vi) −*x*, −*y*+2, −*z*; (vii) *x*, *y*+1, *z*; (viii) *x*, *y*−1, *z*.
